# Mitochondrial defects in the cAMP-PKA-DRP1 pathway in a *Crppa* deletion model of dystroglycanopathy

**DOI:** 10.1242/dmm.052890

**Published:** 2026-09-17

**Authors:** Jihang Luo, Yidan Liu, Danyu Song, Shiqi Yang, Xiaona Fu, Lin Ge, Cuijie Wei, Liya Cui, Yanwei He, Jin Xu, Qiang Shen, Yuxuan Guo, Motoi Kanagawa, Tatsushi Toda, Jingmin Wang, Hong Zhang, Hui Xiong

**Affiliations:** ^1^ Children's Medical Center, Peking University First Hospital, No.5 Le Yuan Road, Daxing District, Beijing 102627, China; ^2^ Department of Neurology, Beijing Children's Hospital, Capital Medical University, National Center for Children's Health, Beijing 100045, China; ^3^ State Key Laboratory of Vascular Homeostasis and Remodeling, The Institute of Cardiovascular Sciences, School of Basic Medical Sciences, Peking University Health Science Center, Beijing 100191, China; ^4^ Laboratory of Electron Microscopy, Peking University First Hospital, Beijing 102600, China; ^5^ Department of Cell Biology and Molecular Medicine, Ehime University Graduate School of Medicine, Shitsukawa 454, Toon, Ehime 791-0295, Japan; ^6^ Department of Neurology, Graduate School of Medicine, The University of Tokyo, 7-3-1 Hongo, Bunkyo-ku, Tokyo 113-8655, Japan

**Keywords:** *CRPPA*, Dystroglycanopathies, Mouse model, Mitochondrial, cAMP-PKA-DRP1

## Abstract

Dystroglycanopathies (DGPs) are autosomal recessive muscular dystrophies caused by abnormal α-dystroglycan glycosylation. *CRPPA* is one causative gene, with deletion of exons 6-9 identified as a founder variant in Chinese patients. Our previous study revealed mitochondrial abnormalities in patient muscle biopsies, although the underlying mechanism(s) remained unclear. A *Crppa* knockout mouse (dy^C^/dy^C^) was generated based on the founder variant, displaying muscle weakness, cerebellar hypoplasia, retinal abnormalities and neonatal lethality within 24 h. Electron microscopy showed mitochondrial structural defects in skeletal muscle, consistent with patient findings. RNA sequencing revealed dysregulation of the cAMP-PKA pathway, accompanied by decreased ATP and reduced phosphorylation of PKA and DRP1 (Ser637). To verify the link between CRPPA deficiency and mitochondrial dysfunction, *Crppa* knockdown C2C12 cells and *CRPPA*-related DGP patient-derived fibroblasts were examined. Both models exhibited reduced DRP1 Ser637 phosphorylation and ATP levels. Treatment with cAMP-PKA activators restored DRP1 phosphorylation and ATP production in a time-dependent manner. Recovery of mitochondrial membrane potential was confirmed by JC-1 staining. These findings suggest that CRPPA deficiency is associated with mitochondrial dysfunction involving the cAMP-PKA-DRP1 axis, suggesting a candidate pathway warranting further investigation for DGPs.

## Teaser

**Summary:** A knockout mouse model reveals that *CRPPA* deficiency is associated with mitochondrial dysfunction involving the cAMP-PKA-DRP1 pathway, identifying a candidate pathway warranting further preclinical investigation for a currently untreatable form of muscular dystrophy.

## INTRODUCTION

Dystroglycanopathies (DGPs) are a group of rare genetic disorders characterized by defects in α-dystroglycan (α-DG) glycosylation, resulting in variable clinical presentations ranging from severe Walker–Warburg syndrome to milder limb-girdle muscular dystrophy (Song et al., 2021; Kanagawa, 2021; Saito, 1993). To date, variants in 19 distinct genes have been identified as causative of DGPs (Song et al., 2021; Kanagawa and Toda, 2017). Among these, *CRPPA* [Online Mendelian Inheritance in Man (OMIM) 614631] encodes cytidine diphosphate-(CDP-)L-ribitol pyrophosphorylase A, which contains an N-terminal catalytic domain and a C-terminal non-catalytic domain (Vuillaumier-Barrot et al., 2012). *CRPPA* variants have been associated with a broad spectrum of DGP phenotypes, yet the molecular mechanisms underlying disease pathogenesis remain incompletely understood. Three kinds of animal models have been developed to investigate CRPPA function. A skeletal muscle-specific conditional knockout mouse model was used to study CDP-ribitol biosynthesis and evaluate CDP-ribitol prodrug therapy (Tokuoka et al., 2022). A systematic *Crppa* knockout mouse model focused on retinal developmental abnormalities and axonal guidance mechanisms (Clements et al., 2017; Wright et al., 2012), but did not address its myopathy and brain structural abnormalities. Additionally, *crppa* knockdown (KD) in zebrafish recapitulated hydrocephalus, reduced eye size and muscle degeneration (Roscioli et al., 2012). Although these models have provided valuable insights, none were designed based on patient-specific variants, and the downstream signaling pathways affected by CRPPA deficiency have not been systematically characterized.

In our recent study, deletion of exons 6-9 in *CRPPA* was identified as a founder variant in the Chinese population through clinical and genetic analysis of 16 patients (Luo et al., 2025). This out-of-frame mutation is predicted to result in premature translation termination and loss of the C-terminal domain. Notably, mitochondrial abnormalities were observed in patient muscle biopsies (Luo et al., 2025). Based on these findings, we utilized CRISPR-Cas9 gene-editing technology to specifically delete exons 6-9 of the *Crppa* gene, creating a novel *Crppa* knockout mouse model for *CRPPA*-related DGP, named ‘dy^C^/dy^C^’. The dy^C^/dy^C^ mice exhibited severe multisystem phenotypes, including muscle weakness, cerebellar hypoplasia and retinal abnormalities, recapitulating the most severe DGPs manifestations in patients. Electron microscopy observations revealed mitochondrial structural abnormalities in skeletal muscle, consistent with patient manifestations. RNA-sequencing analysis identified dysregulation of the cyclic adenosine monophosphate (cAMP)-protein kinase A (PKA) signaling pathway, accompanied by decreased ATP levels and reduced phosphorylation of dynamin-related protein 1 (DRP1) at Ser637, a key factor in mitochondrial fission and dynamics regulation.

To further examine whether CRPPA deficiency is associated with mitochondrial dysfunction involving the cAMP-PKA-DRP1 axis, this study simultaneously employed a *Crppa* KD C2C12 cell model and skin fibroblasts derived from patients with *CRPPA*-related DGPs. Both cell models successfully reproduced α-DG glycosylation deficiency and reduced phosphorylation of PKA and DRP1. Treatment with cAMP-PKA activators – 3-isobutyl-1-methylxanthine (IBMX; 400 μM), a phosphodiesterase inhibitor that prevents cAMP degradation, and 8-bromoadenosine 3′,5′-cyclic monophosphate (8-Br-cAMP; 400 μM), a stable cAMP analog that directly activates PKA – was performed at 24, 48 and 72 h timepoints. In both C2C12 and patient fibroblast models, treatments restored DRP1 phosphorylation, mitochondrial membrane potential and ATP production in a time-dependent manner, with 8-Br-cAMP showing more pronounced effects and optimal rescue observed at 48 h. These results suggested that CRPPA deficiency is associated with mitochondrial dysfunction involving the cAMP-PKA-DRP1 axis in *in vivo* and *in vitro* models. Given the lack of effective treatments for DGPs, our findings identify this pathway as a promising therapeutic target and provide a foundation for developing mechanism-based therapies that may be applicable to a broader range of DGPs.

## RESULTS

### dy^C^/dy^C^ mice: a novel mouse model for severe early-onset *CRPPA*-related DGPs

The deletion of exons 6-9 was identified as a potential founder variant in the Chinese population (Luo et al., 2025). This deletion resulted in an out-of-frame mutation and premature translation termination, predictably leading to the complete absence of the C-terminal domain of the CRPPA protein (Luo et al., 2025). Based on this finding, a mouse model – dy^C^/dy^C^ – was generated through targeted deletion of exons 6-9 of *Crppa* using CRISPR-Cas9 gene-editing technology. Two sgRNAs were designed targeting introns 5 and 9 of the *Crppa* gene to delete 47.5 kb DNA, including exons 6-9 (Fig. 1A). PCR analysis of DNA was used to identify the genotypes of the mice. Three primers – *Crppa*-F (before the deleted region), *Crppa*-R1 (within the deleted region) and *Crppa*-R2 (after the deleted region) – were designed for genotype identification (Fig. 1A). A 506-bp fragment was identified as wild type (WT), a 717-bp fragment was identified as homozygous (Hom; dy^C^/dy^C^), and 506-bp and 717-bp fragments were identified as heterozygous (Het) (Fig. 1B). Sanger sequencing of the PCR product confirmed the deletion of exons 6-9 in *Crppa* in dy^C^/dy^C^ mice (Fig. 1C). At the RNA level, reverse transcription quantitative PCR (RT-qPCR) analysis revealed that *Crppa* transcripts detected by C-terminal-specific primers were significantly reduced in dy^C^/dy^C^ mice, while N-terminal transcripts showed a decreasing trend without this reaching statistical significance (Fig. 1D). This pattern is consistent with nonsense-mediated mRNA decay triggered by the premature termination codon resulting from the out-of-frame deletion. Western blot analysis of the skeletal muscle lysate confirmed the successful model construction. The results showed that the expression level of the C-terminal of Crppa in dy^C^/dy^C^ mice was significantly decreased, as expected (Fig. 1E,F). Notably, the expression level of the N-terminal of Crppa was also found to be decreased (Fig. 1E,F).

**Fig. 1.**
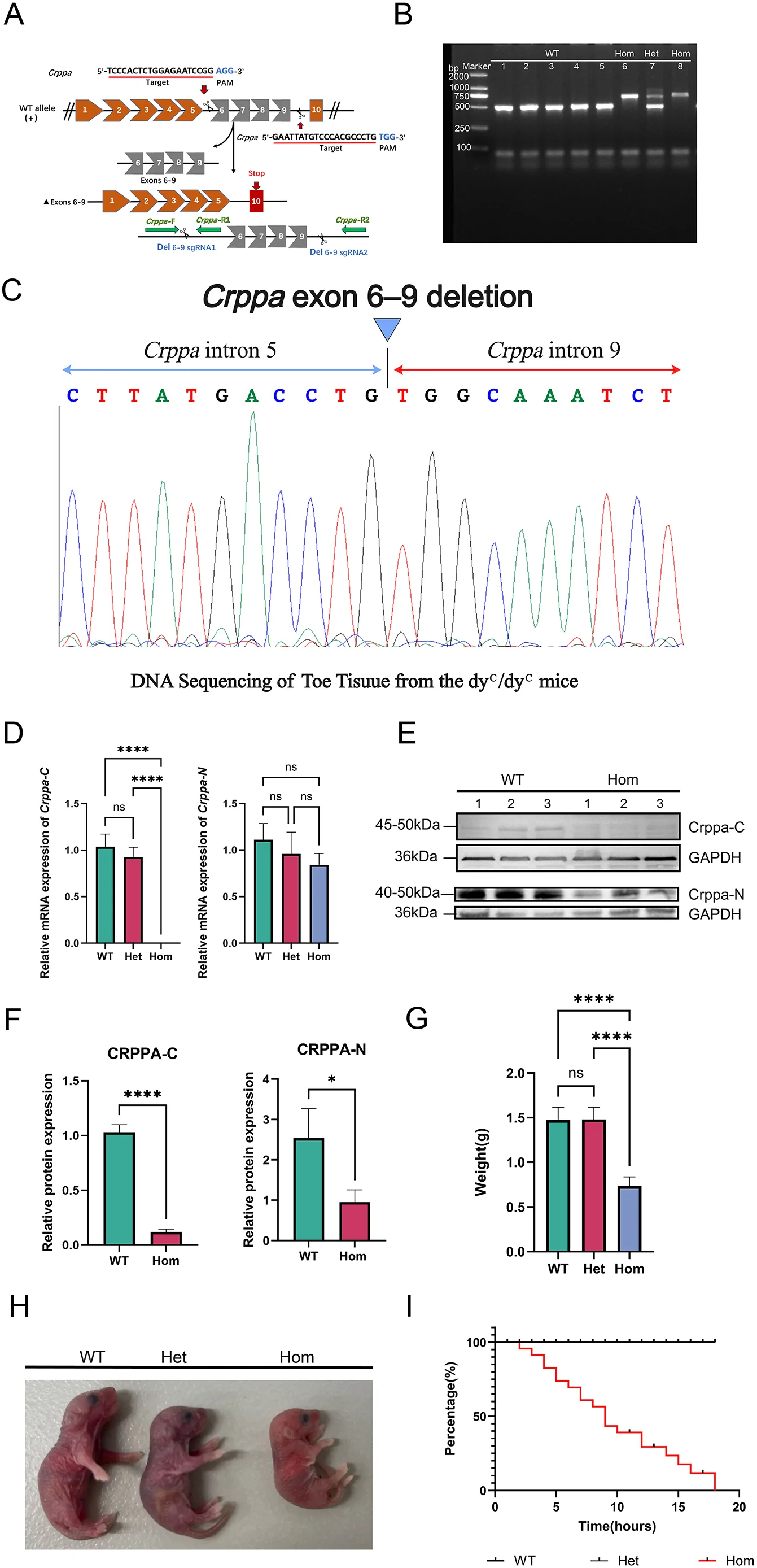
**General phenotype of the *Crppa* knockout mouse model – dy^C^/dy^C^ mice.** (A) Strategy for generating the dy^C^/dy^C^ mice using CRISPR-Cas9 technology. In the *Crppa* gene sequence, the parts marked in orange indicate the target sequences for sgRNA, while the parts marked in green indicate the positions of the PCR primers (*Crppa*-F, *Crppa*-R and *Crppa*-R2; a 506-bp fragment was identified as WT, a 717-bp fragment was identified as Hom, and 506-bp and 717-bp fragments were identified as Het). (B) PCR analysis was used to identify the genotype of F2 mice. (C) Sanger sequencing confirmation of the *Crppa* exons 6-9 deletion. DNA sequencing from toe tissue of dy^C^/dy^C^ mice demonstrates the precise junction between *Crppa* intron 5 and intron 9, confirming successful deletion of exons 6-9. (D) RT-qPCR analysis was performed on muscle samples, confirming the deletion of the C-terminal region of *Crppa* mRNA (*n*=3; ns, *P*>0.05; ****P<0.0001, by one-way ANOVA). (E) Western blot analysis of Crppa protein expression: Crppa-C expression (top) was significantly decreased and Crppa-N expression (bottom) was reduced in muscle samples of Hom mice compared to those of WT mice. (F) The western blot results were quantified using a gray-value analysis, and the results showed a statistically significant difference in Crppa-C (left) and Crppa-N (right) expression between the dy^C^/dy^C^ and WT mice (*n*=3, *P<0.05, ****P<0.0001, by unpaired two-tailed *t*-test). (G) There was no significant difference in body weight between WT and Het mice (*n*=20; ns, *P*>0.05; by Kaplan–Meier); however, dy^C^/dy^C^ mice had significantly lower body weight than that of WT and Het mice (*n*=20, ****P<0.0001, by one-way ANOVA). (H) At 2 h after birth, the dy^C^/dy^C^ mice were noticeably smaller than the WT and Het mice. (I) There was no significant difference in survival rates between WT and Het mice. During the observation period, no deaths of WT (*n*=20) or Het (*n*=20) mice occurred. The dy^C^/dy^C^ mice (*n*=20) had a minimum survival time of 2 h and a maximum survival time of 18 h. Crppa-C, C-terminal region of Crppa; Crppa-N, N-terminal region of Crppa; Het, heterozygous; Hom, homozygous (dy^C^/dy^C^); PAM, protospacer adjacent motif; ns, not significant; RT-qPCR, reverse transcription quantitative PCR; WT, wild type.

Among the offspring of heterozygous crosses, approximately one-quarter were Hom (dy^C^/dy^C^) mice, indicating that the embryos were viable and could be born full term. Compared with WT mice, the dy^C^/dy^C^ mice were significantly smaller and had a significantly lower body weight (*P*<0.0001) (Fig. 1G,H). Severe muscle weakness and a sharp reduction in lifespan were observed, with all the dy^C^/dy^C^ mice (*n*=20) dying within 24 h after birth (median lifespan, 8.5 h; range, 2-18 h) (Fig. 1I). In contrast, the WT and Het mice remained healthy throughout the 1-year observation period. The dy^C^/dy^C^ mice almost completely lost their ability to move after birth, in sharp contrast to their WT and Het littermates (Movie 1).

### Muscle pathology and α-DG glycosylation deficiency in dy^C^/dy^C^ mice

Skeletal muscle, heart, brain and eyes from WT and dy^C^/dy^C^ mice were examined post birth. Hematoxylin and Eosin (HE) staining of skeletal muscle revealed that muscle fibers in both genotypes were immature and interspersed with considerable interstitial tissue. However, dy^C^/dy^C^ mice exhibited disorganized muscle fiber arrangement, more pronounced interstitial tissue expansion and reduced muscle fiber density (*P*<0.05, Fig. 2). Cardiac tissue showed no significant structural abnormalities by HE staining, and Masson staining revealed no statistically significant difference in fibrosis between genotypes (*P*>0.05), suggesting that cardiac defects were not the primary cause of death (Fig. 2). Brain examination revealed cerebellar and brainstem dysplasia in dy^C^/dy^C^ mice. Retinal analysis showed disruptions in the outer plexiform layer and retinal pigment epithelium in dy^C^/dy^C^ mice, contrasting with normal retinal structure in WT mice (Fig. 2).

**Fig. 2.**
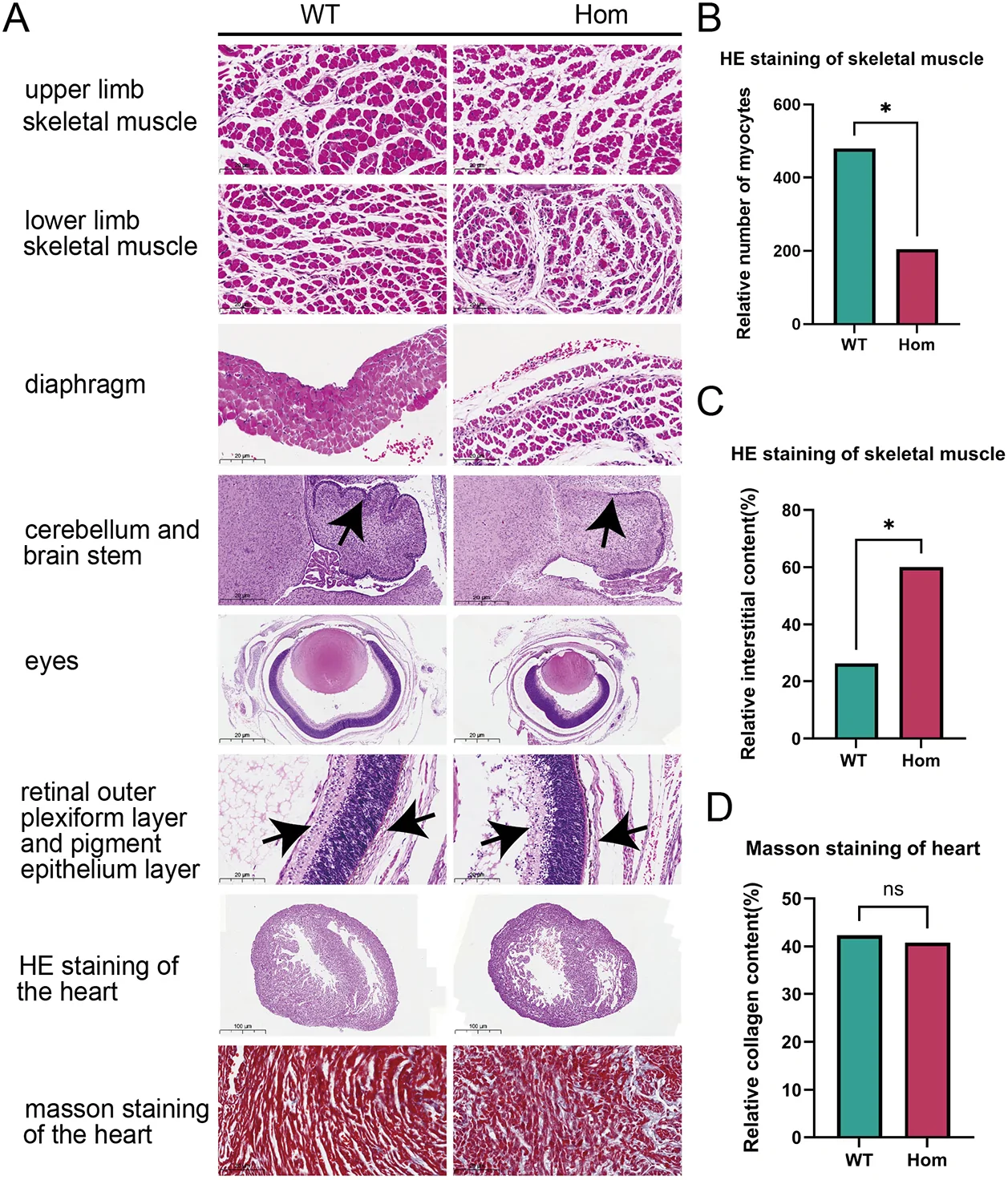
**Muscle pathology of dy^C^/dy^C^ mice.** (A) HE staining revealed that the limb skeletal muscles and diaphragm muscle fibers of WT and dy^C^/dy^C^ mice were immature, disorganized and accompanied by increased interstitial tissue. Cardiac tissue HE staining showed incomplete tissue density in the hearts of the dy^C^/dy^C^ mice, and Masson staining indicated mild fibrosis in the hearts of the dy^C^/dy^C^ mice. HE staining of the brain revealed structural abnormalities in the cerebellum and brainstem of the dy^C^/dy^C^ mice (black arrows). HE staining of eyes indicated significant damage to the outer retinal ganglion cell layer and retinal pigment epithelium in the dy^C^/dy^C^ mice (black arrows). (B,C) Quantification of myocyte numbers confirmed a significant reduction in the dy^C^/dy^C^ mice compared to those in WT controls (B), consistent with the increased interstitial tissue observed in A. The increase in interstitial tissue was more pronounced in the dy^C^/dy^C^ mice (C), with a notable reduction in muscle fibers (*n*=3; three consecutive sections were cut from each mouse, and three random areas were selected from each section for statistical analysis; *P<0.05, by unpaired two-tailed *t*-test). (D) Masson staining (blue) of the heart tissue showed mild fibrosis in the dy^C^/dy^C^ mice, but no significant statistical difference was observed compared to WT mice (*n*=3; ns, *P*>0.05, by unpaired two-tailed *t*-test). HE, Hematoxylin and Eosin.

The glycosylation defect of α-DG was identified by immunofluorescence analysis. In skeletal muscle, the IIH6 antibody (recognizing mature α-DG glycosylation) immunoreactivity was significantly decreased in the dy^C^/dy^C^ mice (*P*<0.05; Fig. 3A,B). In the cerebellum, brainstem and retinal regions, IIH6 immunoreactivity was decreased in the structurally abnormal areas, suggesting that the α-DG glycosylation defect is associated with these pathological changes (Fig. 3C,D).

**Fig. 3.**
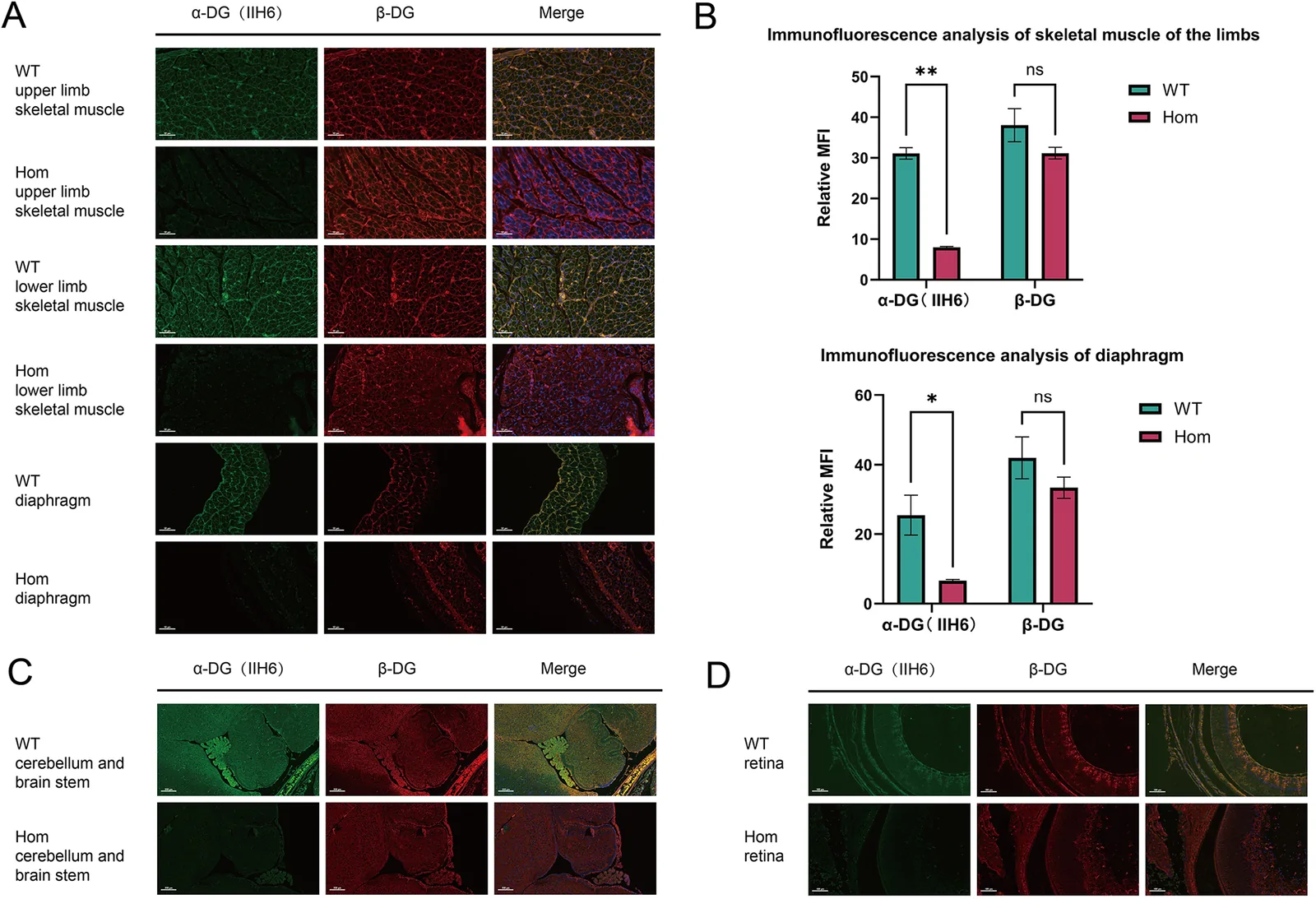
**Immunofluorescence staining of dy^C^/dy^C^ mice**. (A) Immunofluorescence staining of skeletal muscles and diaphragm in the dy^C^/dy^C^ mice indicated marked deficiency in α-DG glycosylation. Scale bars: 50 μm. (B) The fluorescence intensities of immunofluorescence staining of skeletal muscles in the limbs (data of upper- and lower-limb muscles were combined for statistical analysis) and diaphragm were statistically analyzed. The results showed a significant difference in the deficiency in α-DG glycosylation between the dy^C^/dy^C^ mice and WT mice (*n*=3; three consecutive sections were cut from each mouse, and three random areas were selected from each section for statistical analysis; ns, *P*>0.05; *P<0.05, **P<0.01, by two-way ANOVA). (C) Immunofluorescence results of the brain showed deficiency in α-DG glycosylation in the cerebellar brainstem of dy^C^/dy^C^ mice compared to that in WT mice. Scale bars: 200 μm. (D) Immunofluorescence results of the eyes showed deficiency in α-DG glycosylation in the retinal area of dy^C^/dy^C^ mice compared to that in WT mice. Scale bars: 100 μm. DG, dystroglycan; MFI, mean fluorescence intensity.

Membrane proteins were enriched from skeletal muscle extracts using wheat germ agglutinin (WGA) microspheres, and western blot analysis was performed using antibodies against matriglycan (IIH6), α-DG core protein and dystroglycan, along with laminin overlay experiments (Fig. 4). The results showed that the dy^C^/dy^C^ mice presented significantly decreased IIH6 immunoreactivity (*P*<0.05), reduced α-DG core protein molecular mass and weakened laminin binding activity (Fig. 4), indicating that the α-DG glycosylation defect leads to impaired extracellular matrix interactions.

**Fig. 4.**
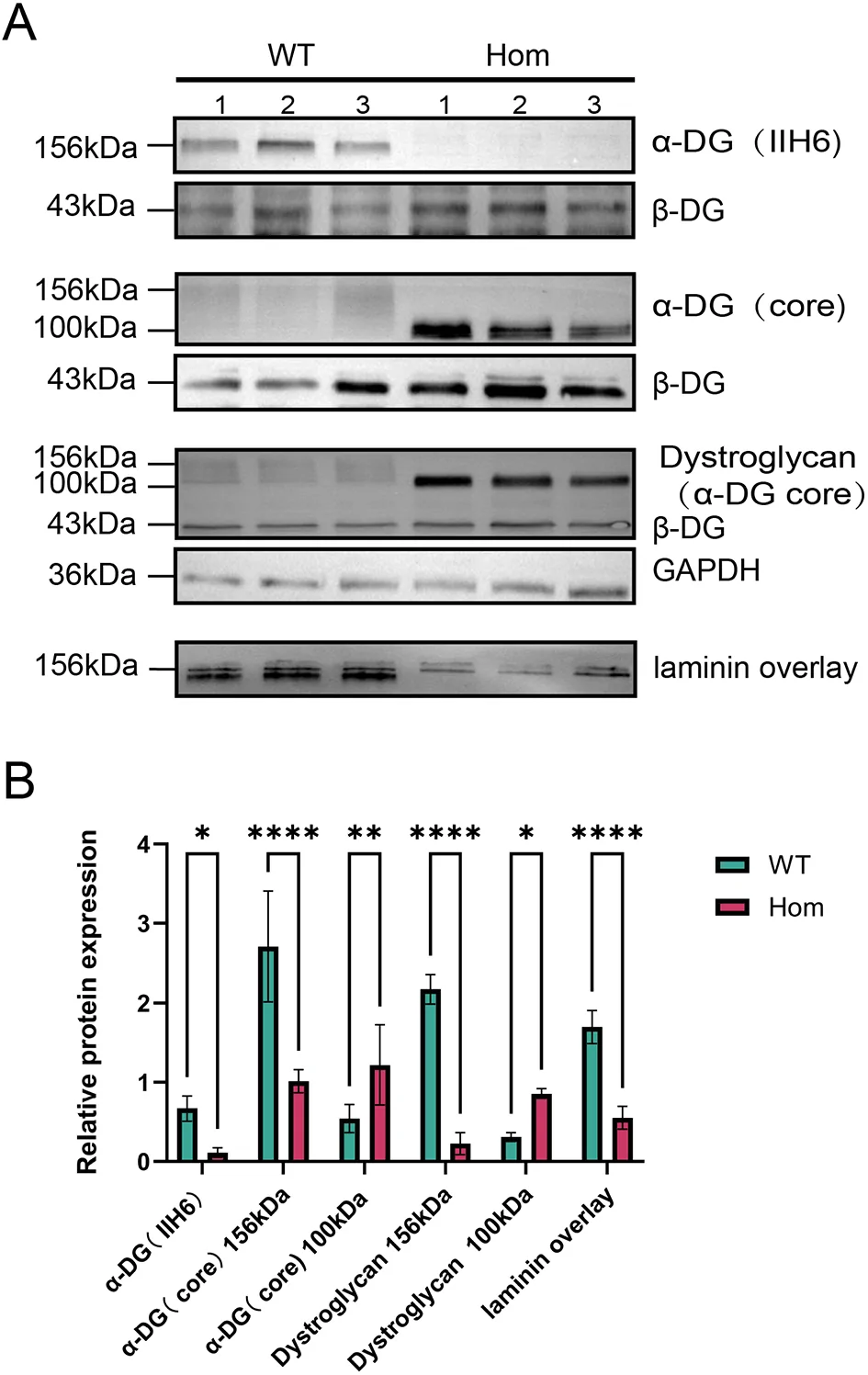
**Deficiency in α-DG glycosylation and laminin binding in dy^C^/dy^C^ mice.** (A) Western blot analysis of WGA-enriched membrane proteins of skeletal muscle from WT and dy^C^/dy^C^ mice. α-DG glycosylation was measured using antibodies against matriglycan (IIH6), α-DG core protein and dystroglycan (recognizes α-DG core protein and β-DG simultaneously), and laminin overlay assay. β-DG and GAPDH were used as controls. (B) The western blot results were quantified using a gray-value analysis, and the results showed significantly reduced IIH6 immunoreactivity, decreased molecular mass of α-DG core protein and impaired laminin-binding activity in the dy^C^/dy^C^ mice compared to that in WT mice (*n*=3, *P<0.05, **P<0.01, ****P<0.0001, by two-way ANOVA). WGA, wheat germ agglutinin.

### Deletion of exons 6-9 in *Crppa* causes mitochondrial dysfunction in dy^C^/dy^C^ mice

Given the previously observed mitochondrial abnormalities in muscle biopsy specimens from patients with *CRPPA*-related DGPs, this study employed electron microscopy for ultrastructural analysis to further explore the cellular mechanisms underlying the pathology of *Crppa*-deficient mice (Luo et al., 2025). Examination of skeletal muscle, cardiac muscle tissue, brain tissue and ocular structures revealed significant mitochondrial morphological abnormalities in the dy^C^/dy^C^ mice, including swelling, increased volume and disordered distribution patterns. Structural defects included damage to the inner and outer mitochondrial membranes, disruption of mitochondrial cristae structure and mitochondrial vacuolization (Fig. 5A, white arrows). Consistent with these structural abnormalities, biochemical analysis showed a significant reduction in ATP levels in the skeletal muscle of the dy^C^/dy^C^ mice compared to those in the skeletal muscle of WT mice (*P*<0.001; Fig. 5B).

**Fig. 5.**
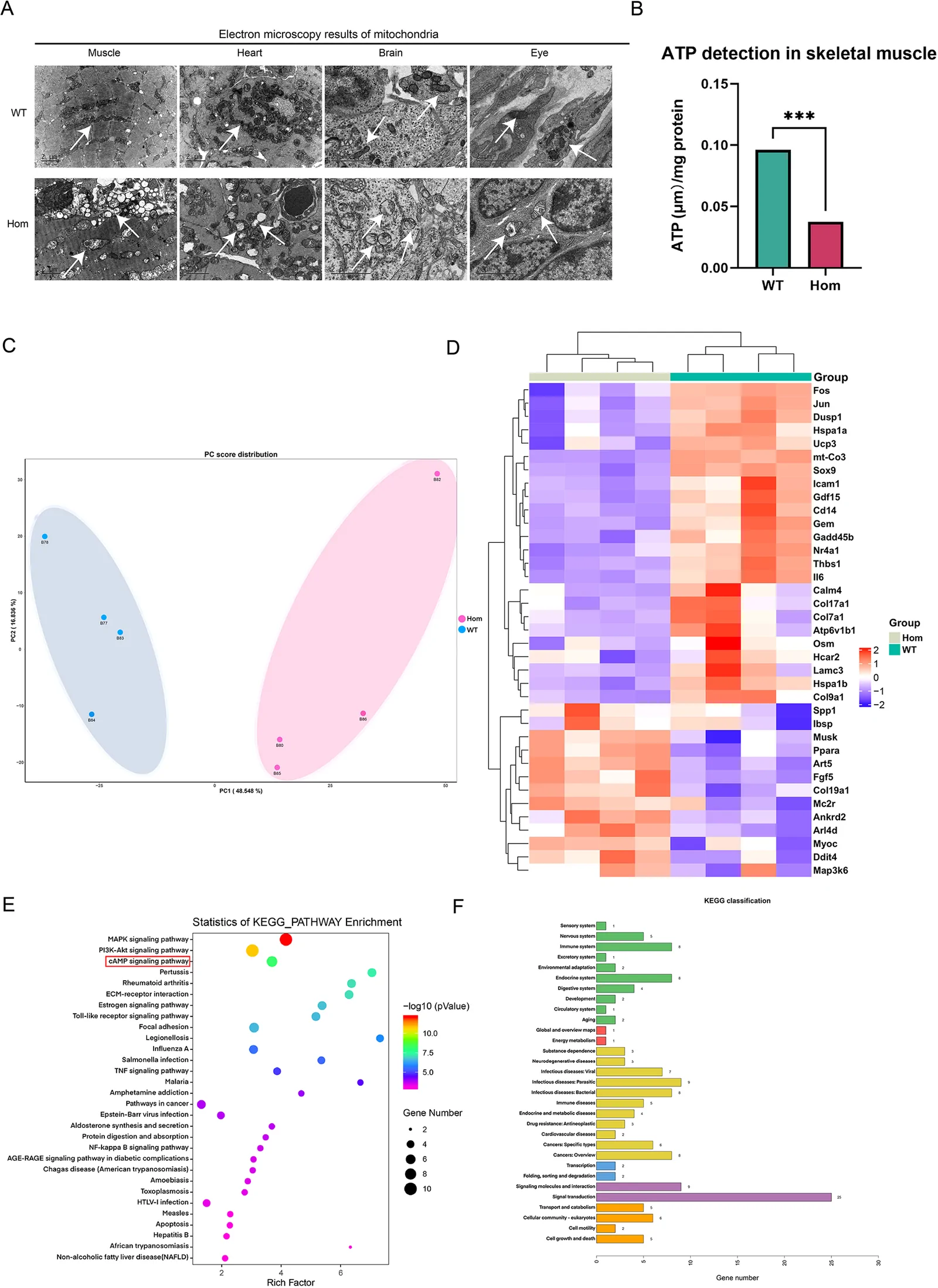
**Deletion of exons 6-9 in *Crppa* leads to mitochondrial abnormalities and transcriptional dysregulation.** (A) Electron microscopy of the skeletal muscle in the lower limbs, cardiac tissue, brain tissue and ocular tissue of mice revealed mitochondrial structural abnormalities in the dy^C^/dy^C^ mice compared to WT mice. These abnormalities included morphological irregularities in the muscle fibers of the dy^C^/dy^C^ mice, manifesting as swelling, increased volume and a disordered distribution pattern. At the mitochondrial level, severe structural defects were evident. Both the inner and outer mitochondrial membranes sustained damage, and the mitochondrial cristae were disrupted (white arrows). Scale bars: 2 μm. (B) ATP levels were measured in fresh skeletal muscle tissue from mice, and the results indicated that the ATP concentration in the dy^C^/dy^C^ mice was significantly lower than that in WT mice (both biological replicates and technical replicates were set at *n*=3; ***P<0.001, by unpaired two-tailed *t*-test). (C) Principal component analysis of dimensionality reduction of differentially expressed genes in lower-limb skeletal muscle: a comparative study of the dy^C^/dy^C^ mice against WT controls (*n*=4). (D) Compared to those in WT muscle, differentially expressed genes (|log2FC|≥1, *P*<0.05) in the dy^C^/dy^C^ mouse muscle showed distinct patterns in cell growth, development, differentiation, regeneration, metabolism and mitochondrial function. (E) KEGG pathway enrichment analysis revealed distinctions: the dy^C^/dy^C^ mouse muscle, relative to WT muscle, exhibited altered patterns in cell growth and biosynthesis, metabolism and mitochondrial function. (F) KEGG classification: compared to WT mice, the dy^C^/dy^C^ mice showed the highest number of differentially expressed genes in signaling, metabolism and nervous system pathways. KEGG, Kyoto Encyclopedia of Genes and Genomes; PC, principal component.

### RNA sequencing identifies cAMP-PKA-DRP1 axis dysregulation as a mechanism underlying mitochondrial dysfunction

To explore the molecular mechanism of mitochondrial dysfunction, RNA sequencing was performed on the lower-limb skeletal muscle tissues of WT and dy^C^/dy^C^ mice. Principal component analysis revealed significant separation among different genotypes (Fig. 5C). Differential expression analysis identified 459 differentially expressed genes in the muscles of the dy^C^/dy^C^ mice, including 189 upregulated genes and 270 downregulated genes (Fig. 5D). Kyoto Encyclopedia of Genes and Genomes (KEGG) pathway enrichment analysis revealed significant alterations in multiple signaling pathways (Fig. 5E). Notably, the cAMP-PKA signaling pathway, which regulates mitochondrial dynamics through phosphorylation of DRP1 at Ser637, was identified as one of the significantly dysregulated pathways (*P*<0.05). This finding suggested a potential mechanistic link between Crppa deficiency and mitochondrial dysfunction. In addition to the cAMP-PKA signaling pathway, KEGG analysis identified dysregulation of the MAPK signaling pathway, calcium signaling pathway and extracellular matrix (ECM)-receptor interaction pathway. The altered ECM-receptor interaction likely reflects the direct consequence of α-DG glycosylation deficiency, while changes in MAPK and calcium signaling may represent secondary responses to membrane instability. The cAMP-PKA pathway was prioritized for mechanistic follow up, given its established role in regulating mitochondrial dynamics via DRP1 Ser637 phosphorylation. KEGG functional classification analysis (Fig. 5F) showed significantly differentially expressed genes in signaling (25 genes), nervous system (five genes) and immune system (eight genes) pathways. To validate these findings, we conducted targeted RT-qPCR analysis of key genes in the cAMP-PKA pathway in skeletal muscle tissues. The results showed a consistent pattern of differential expression in the dy^C^/dy^C^ mice (*Jun*, *P*<0.001; *Fos*, *P*<0.01; *Hcar2*, *P*<0.05; *Mc2r*, *P*<0.05; *Sox9*, *P*<0.05; *Calm4*, *P*<0.001; Fig. 6A). Western blot analyses further revealed a marked reduction in PKA phosphorylation in the dy^C^/dy^C^ mice (*P*<0.05). Given that PKA regulates mitochondrial division mainly by phosphorylating DRP1 at Ser637, the phosphorylation status of this residue was examined, and the results confirmed that the level of phosphorylated DRP1 (Ser637) was significantly reduced in the mutant tissues (*P*<0.05; Fig. 6B). Notably, expression profiles of other key mediators of mitochondrial dynamics remained largely unaltered between genotypes [*Tom20* (also known as *Tomm20*), *Opa1*, *DRP1* (also known as *Dnm1l*), *Mff*, *Mfn1*; *P*>0.05; Fig. S1]. These results indicate that Crppa deficiency specifically impairs the cAMP-PKA-DRP1 axis.

**Fig. 6.**
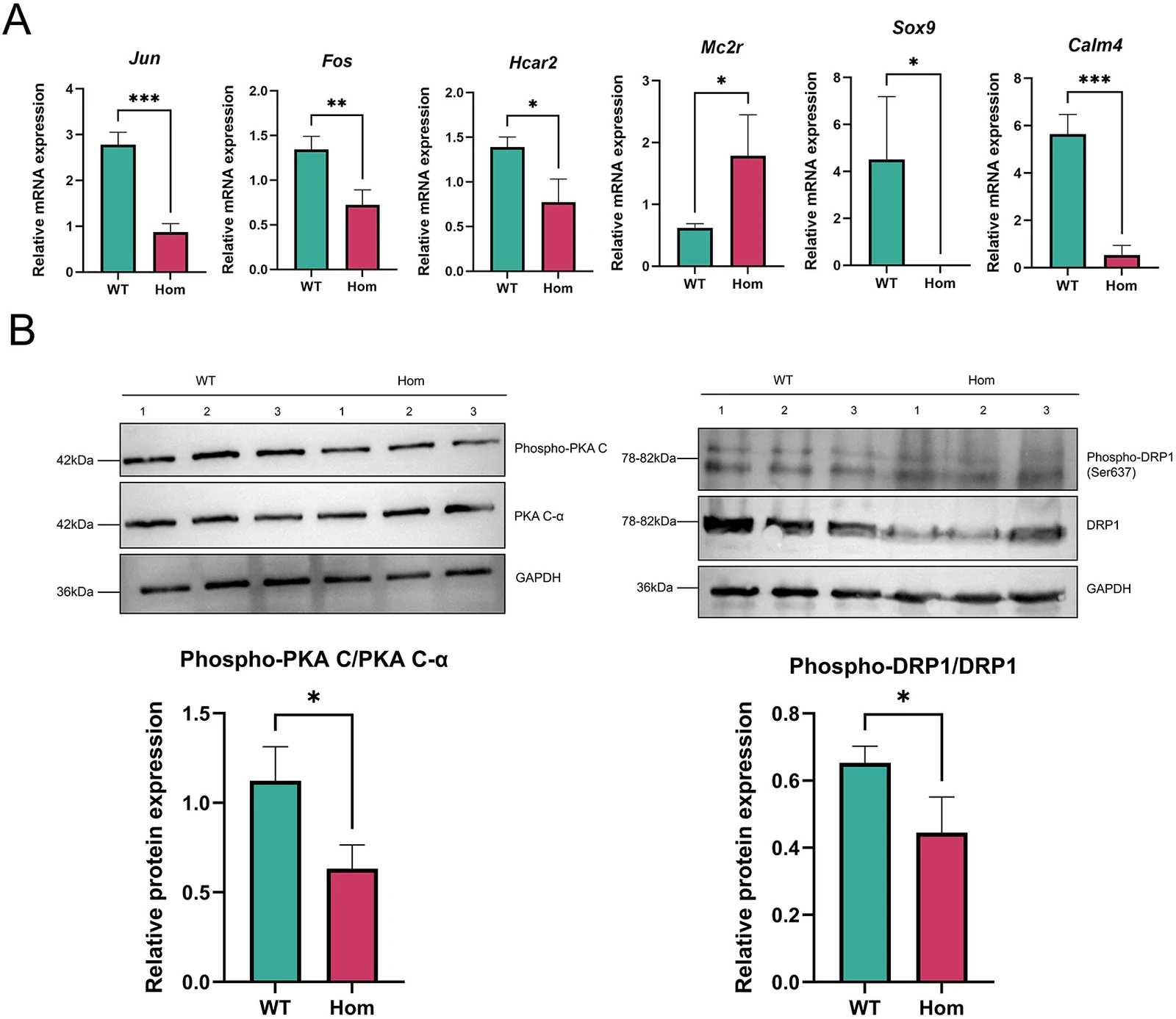
**Crppa deficiency impairs cAMP-PKA signaling and DRP1 phosphorylation in skeletal muscle.** (A) RNA was extracted from the skeletal muscle of the mouse lower limbs, and RT-qPCR analysis was used to examine the expression of key cAMP-PKA pathway genes (*n*=3; *P<0.05, **P<0.01, ***P<0.001, by unpaired two-tailed *t*-test). (B) Reduced phosphorylation of PKA and DRP1 in skeletal muscle of dy^C^/dy^C^ mice. Western blot analysis and quantitative densitometry of phosphorylated and total protein levels in skeletal muscle lysates from WT mice and dy^C^/dy^C^ mice. Western blotting demonstrated significantly reduced phosphorylation of PKA catalytic subunit (phospho-PKA C) relative to total PKA C-α (left), with corresponding reduction in phosphorylation of DRP1 at serine 637 [phospho-DRP1 (Ser637)] relative to total DRP1 (right). Quantitative analyses revealed significant decreases in phospho-PKA C/PKA C-α and phospho-DRP1/DRP1 ratios in the dy^C^/dy^C^ mice compared to those in WT littermates. GAPDH served as a loading control for normalization (*n*=3; *P<0.05, by unpaired two-tailed *t*-test).

### Pharmacological activation of cAMP-PKA pathway rescues mitochondrial dysfunction in *Crppa* KD cells and patient-derived fibroblasts

To investigate the mechanistic association between CRPPA deficiency and mitochondrial dysfunction involving the cAMP-PKA-DRP1 axis, a *Crppa* KD C2C12 cell model and patient-derived human dermal fibroblasts derived from a patient with *CRPPA* variants were employed. Cells transfected with non-targeting control sequences served as negative controls (NCs) for both cell types. Western blot analysis confirmed successful KD of Crppa protein expression in C2C12 cells, while patient fibroblasts exhibited loss of functional CRPPA protein (Fig. 7A,B). Both cell models showed reduced α-DG glycosylation, as detected by IIH6 immunoreactivity (Fig. 7C,D), decreased phosphorylation of PKA and DRP at Ser637 (Fig. 7E,F), and significantly reduced ATP levels, recapitulating the molecular defects observed *in vivo* (Fig. 7G).

**Fig. 7.**
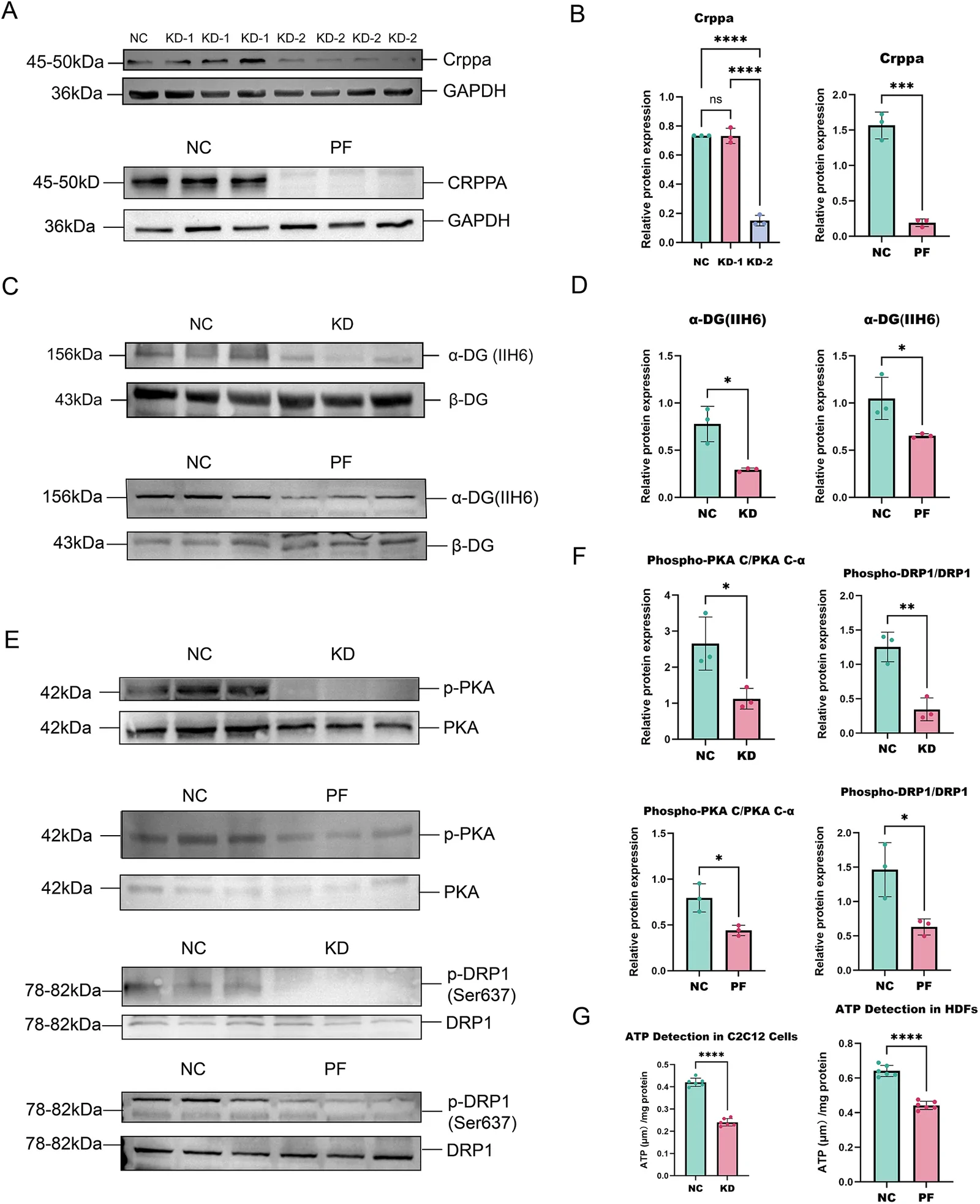
**Establishment and characterization of CRPPA-deficient cellular models in C2C12 myoblasts and patient-derived fibroblasts.** (A,B) Western blot analysis (A) and quantitative densitometry (B) of Crppa protein expression. Upper panels: C2C12 myoblasts transfected with two different siRNAs targeting *Crppa* (KD-1 and KD-2) compared to NC. KD-2 demonstrated more effective knockdown of Crppa expression than did KD-1 (*n*=3; ns, *P*>0.05; ****P<0.0001, one-way ANOVA). Lower panels: patient-derived fibroblasts showed significantly reduced CRPPA expression compared to normal control fibroblasts (NC) (*n*=3; ***P<0.001, by unpaired two-tailed *t*-test). GAPDH served as a loading control. (C) Immunoblot analysis using IIH6 antibody to assess α-DG glycosylation status. Upper panels: *Crppa* knockdown C2C12 cells (using siRNA KD-2) showed reduced α-DG glycosylation compared to that in control cells. Lower panels: patient-derived fibroblasts exhibited similarly decreased IIH6 immunoreactivity compared to that in normal controls. β-DG served as a loading control. (D) Quantitative analysis confirmed significant reduction in functional α-DG glycosylation in both cellular models (*n*=3; *P<0.05, by unpaired two-tailed *t*-test). (E,F) Assessment of cAMP-PKA-DRP1 signaling pathway. Western blot analysis (E) and quantitative densitometry (F) revealed significantly decreased phosphorylation of PKA catalytic subunit (phospho-PKA C/PKA C-α ratio) and DRP1 at serine 637 (phospho-DRP1 Ser637/DRP1 ratio) in both *Crppa* knockdown C2C12 cells and patient-derived fibroblasts compared to their respective controls, indicating impaired mitochondrial dynamics signaling in both models (*n*=3; *P<0.05 and **P<0.01, by unpaired two-tailed *t*-test). (G) ATP concentration measurements. *Crppa* knockdown C2C12 cells (left) and patient-derived fibroblasts (right) demonstrated significantly reduced ATP levels compared to those of their respective controls, indicating compromised mitochondrial energy production (*n*=3; ****P<0.0001, by unpaired two-tailed *t*-test). KD-1, knockdown with siRNA-1; KD-2, knockdown with siRNA-2; NC, negative control; PF, patient-derived fibroblasts; PKA, protein kinase A.

To verify that mitochondrial dysfunction in Crppa-deficient cells is mediated through the cAMP-PKA-DRP1 axis, pharmacological rescue experiments were performed using two complementary approaches: IBMX (400 μM), a non-selective phosphodiesterase inhibitor that elevates intracellular cAMP levels, and 8-Br-cAMP (400 μM), a cell-permeable cAMP analog that directly activates PKA. Both compounds were tested in KD C2C12 cells and PF at 24, 48 and 72 h. Western blot analysis showed that DRP1 Ser637 phosphorylation in both cell types was restored in a time-dependent manner after treatment, with the effect of 8-Br-cAMP being significantly stronger than that of IBMX (Fig. 8A,B). In C2C12 cells, the DRP1 phosphorylation level in the KD+drug group was significantly higher than that in the KD group at all time points (*P*<0.0001). Similarly, in patient fibroblasts, the DRP1 phosphorylation level in the PF+drug (PF, patient-derived fibroblasts) group was significantly restored compared with that in the untreated patient-derived fibroblasts (*P*<0.0001), with the best recovery effect occurring at 48-72 h.

**Fig. 8.**
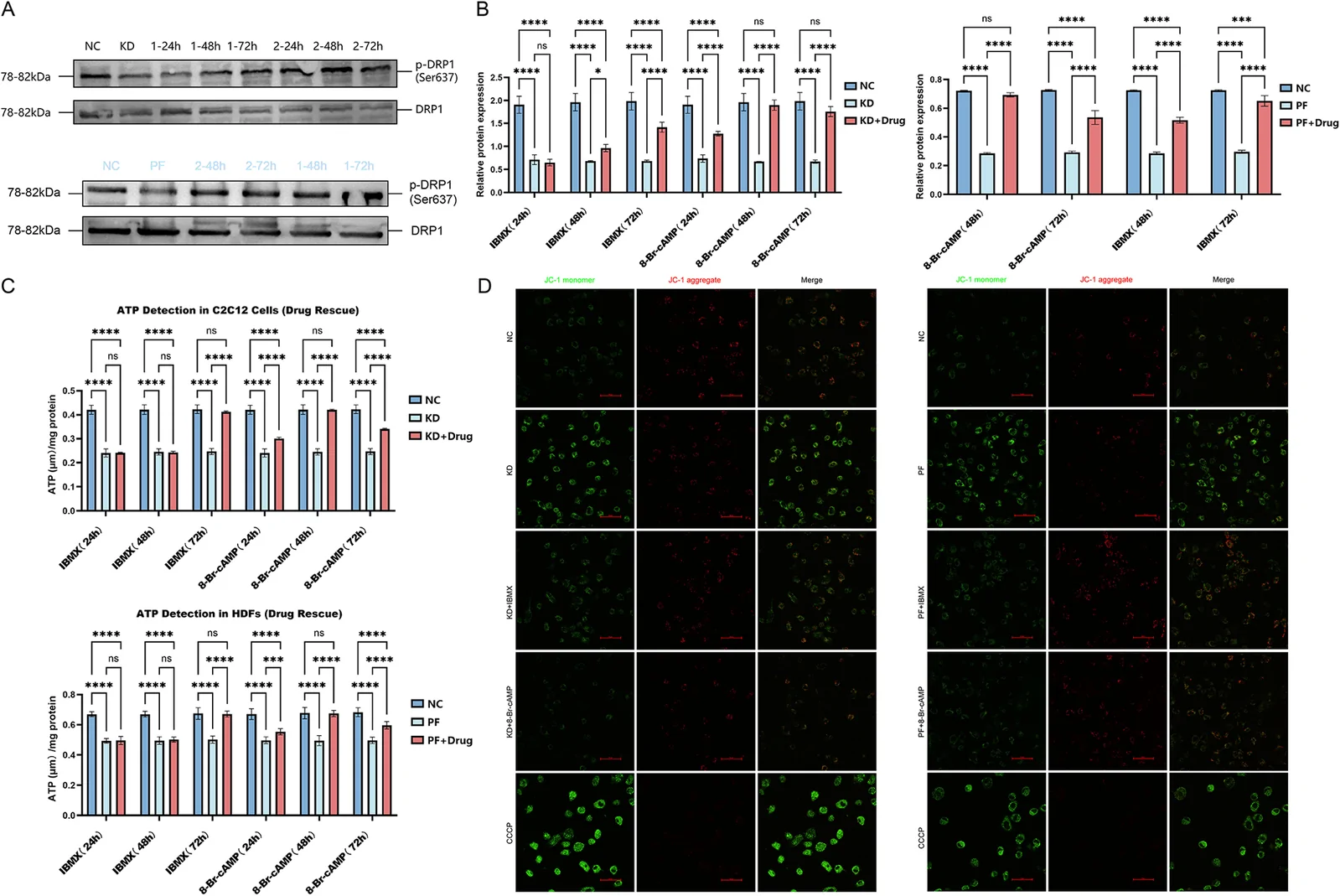
**Pharmacological activation of cAMP-PKA signaling restores DRP1 phosphorylation and mitochondrial function in CRPPA-deficient C2C12 myoblasts and patient-derived fibroblasts.** (A,B) Western blot analysis (A) and quantitative densitometry (B) showing time-dependent restoration of DRP1 Ser637 phosphorylation following treatment with IBMX (drug 1) or 8-Br-cAMP (drug 2) at 24, 48 and 72 h timepoints. Upper panels in A and left graph in B: in C2C12 myoblasts, *Crppa* knockdown cells (KD) exhibited reduced p-DRP1 (Ser637) levels compared to control cells (NC), which were significantly restored following drug treatment (KD+Drug). Lower panels in A and right graph in B: patient-derived fibroblasts (PF) similarly showed decreased p-DRP1 (Ser637) compared to normal control fibroblasts (NC), with significant restoration upon IBMX or 8-Br-cAMP treatment (PF+Drug). Both drugs showed time-dependent rescue effects, with 8-Br-cAMP demonstrating more pronounced restoration. Total DRP1 served as a loading control (*n*=3; ns, *P*>0.05; *P<0.05, ***P<0.001, ****P<0.0001, by one-way ANOVA). (C) ATP concentration measurements under different treatment conditions. Upper panel: C2C12 cells showed significantly reduced ATP levels compared to NC. Treatment with IBMX or 8-Br-cAMP at 24, 48 and 72 h substantially restored ATP content. Lower panel: in HDFs, similar ATP deficits were observed in patient-derived fibroblasts compared to NC, with significant recovery following drug treatment (PF+Drug). Both IBMX and 8-Br-cAMP effectively rescued ATP production in a time-dependent manner (*n*=3; ns, *P*>0.05; ***P<0.001, ****P<0.0001, by one-way ANOVA). (D) JC-1 staining analysis of mitochondrial membrane potential. Representative fluorescence microscopy images showing JC-1 aggregates (red fluorescence, indicating healthy mitochondria with high membrane potential) and JC-1 monomers (green fluorescence, indicating depolarized mitochondria with low membrane potential). Left panels: in C2C12 cells, KD resulted in decreased red/green fluorescence ratio, indicating mitochondrial depolarization. Treatment with IBMX (KD+IBMX) or 8-Br-cAMP (KD+8-Br-cAMP) restored mitochondrial membrane potential toward NC levels. Right panels: patient-derived fibroblasts exhibited similar mitochondrial depolarization, which was effectively rescued by IBMX (PF+IBMX) or 8-Br-cAMP (PF+8-Br-cAMP) treatment. CCCP treatment served as a positive control for complete mitochondrial depolarization in both cell types, showing predominantly green fluorescence with minimal red signal. Scale bars: 50 μm. CCCP, carbonyl cyanide m-chlorophenylhydrazone; HDF, human dermal fibroblast; IBMX, 3-isobutyl-1-methylxanthine; 8-Br-cAMP, 8-bromoadenosine 3′,5′-cyclic monophosphate.

The restoration of DRP1 phosphorylation levels was accompanied by a significant improvement in mitochondrial function. In both cell models, ATP content was significantly restored after treatment (Fig. 8C). In C2C12 cells, the ATP level in the KD group was significantly lower than that in the NC group, while drug treatment significantly increased ATP production, especially after 48 and 72 h of 8-Br-cAMP treatment (*P*<0.0001). Consistent results were observed in patient-derived fibroblasts: compared with untreated patient-derived fibroblasts, the PF+drug group showed significant recovery of ATP levels at all time points (*P*<0.0001). JC-1 staining (which provides a measure of mitochondrial membrane potential) further confirmed the recovery of mitochondrial membrane potential in treated cells (Fig. 8D). In KD C2C12 cells and patient-derived fibroblasts, CRPPA-deficient cells exhibited enhanced green fluorescence (JC-1 monomer) and weakened red fluorescence (JC-1 aggregate), indicating mitochondrial depolarization and cell apoptosis. IBMX or 8-Br-cAMP treatment restored the red/green fluorescence ratio to the control group level, with more complete recovery after 8-Br-cAMP treatment.

## DISCUSSION

DGPs are a group of autosomal recessive genetic disorders caused by defects in the O-linked glycosylation of α-DG. α-DG is a key component for maintaining the integrity of the muscle membrane and the function of the central nervous system (Chen et al., 2021; Ge et al., 2019). Recently, we identified a founder variant of deletion of exons 6-9 in the *CRPPA* gene in the Chinese population, and patients with this variant exhibited severe DGP phenotypes (Luo et al., 2025). To elucidate the disease mechanism, establishing an animal model is crucial. Although all patients carried a heterozygous deletion of exons 6-9 in the *CRPPA* gene and had another pathogenic variant on the second allele, constructing a corresponding compound heterozygous mouse model was still associated with significant technical challenges. We established a mouse model, named dy^C^/dy^C^, with deletion of exons 6-9 in *Crppa*, aiming to explore the pathogenic mechanism of this specific mutation and potential therapeutic strategies. The results showed that the dy^C^/dy^C^ mice, as the first patient-specific mutation model, successfully reproduced the severe phenotypes of *CRPPA*-related DGPs, whereas heterozygous deletion mice did not show obvious phenotypes, suggesting that the disease mechanism involves haploinsufficiency. Western blot analysis revealed that the expression of both the C-terminal and N-terminal proteins of Crppa was decreased in the dy^C^/dy^C^ mice, indicating that C-terminal deletion significantly affected the expression of the N-terminal catalytic domain. This finding suggested that although the C-terminal domain has no direct catalytic activity, it is crucial for maintaining the overall protein stability (Willer et al., 2012; Gerin et al., 2016). Deletion of the C-terminal region likely triggers the degradation of the entire Crppa protein, which explains why patients with C-terminal variations exhibit similar phenotypes to those of patients with N-terminal variants. The previously reported N-terminal knockout *Crppa* mouse model died on day 0 after birth and exhibited extremely severe phenotypes, further supporting this hypothesis (Wright et al., 2012). The similarity between this model and the current C-terminal deletion model strengthens the theory that “C-terminal deletion impairs N-terminal enzyme function through protein instability” (Wright et al., 2012). Notably, although previous *Crppa* mouse models provided valuable insights into specific aspects of disease pathology, including skeletal muscle pathology in conditional knockout mice and retinal development abnormalities in systemic knockout mice, none were based on patient-specific variants identified through clinical genetic analysis (Kanagawa, 2021; Rubio-Fernández et al., 2018). This model fills this gap by precisely reproducing the genetic defect observed in patients, thereby enhancing its translational relevance in therapeutic development.

By comparing and analyzing the dy^C^/dy^C^ mice with WT mice, three characteristic pathological features were discovered: increased interstitial tissue, reduced muscle fiber density and severe α-DG glycosylation deficiency. These observations confirm that the deletion of exons 6-9 of *Crppa* leads to severe DGPs and that the pathological changes are likely to begin during the embryonic development stage. An interesting aspect of this model is that although dy^C^/dy^C^ mice can complete embryonic development, they all die within 24 h after birth. This time pattern can be attributed to the lack of need for autonomous breathing before birth, which results in abnormal diaphragm function not being clinically evident during the embryonic stage. However, the transition to life outside the womb requires spontaneous breathing. The diaphragm defects diagnosed by histopathology will severely impair the respiratory capacity of the dy^C^/dy^C^ mice (Stedman et al., 1991; Huang et al., 2011). The observed phenomena of progressive respiratory failure and feeding intolerance are highly consistent with the end-stage features of severe DGPs in humans. Multiorgan analysis revealed that pathological changes were not limited to skeletal muscle and also included cerebellar and brainstem dysplasia, structural abnormalities of the retina and defects in α-DG glycosylation in all affected tissues. Of note, cardiac tissue showed no significant structural abnormalities or fibrosis, suggesting that cardiac dysfunction was not a major cause of neonatal lethality in this model. This phenotypic fidelity validates the physiological relevance of the dy^C^/dy^C^ model for studying disease pathogenesis while being functionally robust for preclinical treatment evaluation.

In a previous clinical study from this group, mitochondrial abnormalities were observed in muscle biopsies from a patient with *CRPPA*-related DGP, suggesting that mitochondrial dysfunction is involved in disease pathogenesis (Luo et al., 2025). This study, through electron microscopy observations, found that the dy^C^/dy^C^ mice had consistent mitochondrial structural alterations in skeletal muscle, cardiac muscle, brain tissue and eye tissue, manifesting as mitochondrial swelling, membrane damage and destruction of cristae structure, accompanied by a significant decrease in ATP levels. These findings are consistent with clinical observations, indicating that mitochondrial dysfunction is a common pathological feature of the affected organ systems (Hinton et al., 2024; Hughes et al., 2019; Rybalka et al., 2014). This is unexpected because DGPs have traditionally been mainly regarded as structural diseases caused by disruptions in the ECM-membrane interactions (Nickolls et al., 2020; Brancaccio, 2019; Sharaf-Eldin, 2025; Yoshida-Moriguchi and Campbell, 2015). Beyond the structural defects observed by electron microscopy, our functional assessments reveal a coordinated impairment of mitochondrial activity. The reduction in ATP levels reflects compromised oxidative phosphorylation capacity, while the loss of mitochondrial membrane potential detected by JC-1 staining indicates disruption of the electrochemical gradient across the inner mitochondrial membrane. Together, these findings suggest that CRPPA deficiency leads to a state of bioenergetic failure that likely contributes to multiple disease manifestations, including impaired muscle contractility and reduced membrane repair capacity. Future studies employing oxygen consumption rate measurements would further quantify the respiratory deficits described here.

We explored the molecular mechanisms of mitochondrial dysfunction by conducting RNA sequencing on skeletal muscle tissue. The analysis revealed significant alterations in multiple signaling networks, particularly in the cAMP-PKA pathway. Notably, this pathway regulates mitochondrial dynamics by phosphorylating DRP1 at Ser637, thereby inhibiting excessive mitochondrial fission (Tian et al., 2026; Cao et al., 2023; Lin et al., 2025). This discovery reveals the potential mechanistic link between Crppa deficiency and the observed mitochondrial abnormalities. Western blot analysis confirmed reduction in the phosphorylation levels of PKA and DRP1 at the Ser637 site, thereby establishing the molecular connection between Crppa deficiency and mitochondrial dynamic dysfunction. The specificity of this defect is particularly notable: no significant differences were observed in genes encoding other mitochondrial dynamic-related proteins (including *Opa1*, *Mff*, *Mfn1*, and *Tom20*) among different genotypes. This selectivity indicates that the mitochondrial abnormalities reflect a specific disruption of the cAMP-PKA-DRP1 regulatory axis rather than general cellular stress or non-specific mitochondrial dysfunction. Although multiple mechanisms are worth considering (including altered mechanical transduction signals or membrane instability affecting adenylate cyclase activity), the question of how α-DG glycosylation deficiency leads to the disruption of the cAMP-PKA pathway remains incompletely resolved (Kwon et al., 2010; Johnson et al., 2018).

To investigate the mechanistic association between CRPPA deficiency and mitochondrial dysfunction involving the cAMP-PKA-DRP1 axis, this study employed both a *Crppa* KD C2C12 cell model and patient-derived skin fibroblasts. Both cell models successfully reproduced the key molecular and functional defects observed *in vivo*, including α-DG defects, decreased phosphorylation levels of PKA and DRP1, reduced ATP production and mitochondrial dysfunction. Subsequently, pharmacological intervention experiments were conducted using the phosphodiesterase inhibitor IBMX and the cAMP analog 8-Br-cAMP to verify whether activation of the cAMP-PKA pathway could restore mitochondrial function. Both treatments achieved time-dependent recovery of DRP1 Ser637 phosphorylation levels in C2C12 cells and patient fibroblasts (Chen et al., 1998; Lee et al., 2016; Wang et al., 2018; Xie et al., 2024; Ribeiro et al., 2015; Hameed et al., 2018). It is worth noting that 8-Br-cAMP demonstrated a more significant effect than IBMX, suggesting that direct activation of PKA may be more effective than indirectly increasing the level of cAMP through phosphodiesterase inhibition. It should be noted that IBMX and 8-Br-cAMP are not pathway-specific agents, and the observed rescue effects may involve cAMP-dependent effectors beyond PKA. Genetic rescue experiments will be needed to confirm pathway specificity. This difference may reflect the existence of multiple cAMP response pathways, and not all pathways contribute equally to the recovery effect. The restoration of DRP1 phosphorylation was accompanied by significant improvements in multiple parameters in both cell types: ATP content was significantly restored, and JC-1 staining indicated that mitochondrial membrane potential was reconstructed. Time-course experiments showed that the maximum recovery effect was achieved at 48 h, and the effect tended to stabilize after 72 h. This time pattern indicates that the recovery of mitochondrial function requires a process of protein renewal and organelle remodeling, and the plateau period may reflect the establishment of a new steady state or the activation of a negative-feedback mechanism. These pharmacological rescue experiments are consistent with the involvement of the cAMP-PKA-DRP1 axis in mitochondrial dysfunction in CRPPA-deficient cells, supporting the association between this signaling pathway and the observed cellular defects. However, genetic rescue experiments will be required to establish direct causality at the DRP1 Ser637 site. Restoring ATP levels is particularly crucial because ATP depletion is likely to be involved in multiple aspects of the disease pathology, including muscle contractile dysfunction and impaired membrane repair capacity (Wikström and Saari, 1976; Mózsik et al., 2000; Klingenberg, 1975).

Based on these findings, the cAMP-PKA-DRP1 axis represents a candidate pathway warranting further preclinical evaluation for *CRPPA*-related DGPs. Unlike gene therapy or glycosylation enhancement strategies, activating this pathway through small molecules has practical advantages, including the established pharmacological properties of phosphodiesterase inhibitors and cAMP analogs (Phillips et al., 2005; Knebel et al., 2013; Osadchii et al., 2005; Piroozmanesh et al., 2022). Current research indicates that CRPPA deficiency forms a pathological feedback loop: the initial damage to the cAMP-PKA-DRP1 pathway leads to mitochondrial dysfunction and reduced ATP production, which in turn inhibits adenylate cyclase activity and further disrupts cAMP generation, thus creating a self-reinforcing vicious cycle (Bellomo et al., 2018). This mechanism indicates that therapeutic intervention is most effective when initiated at an early stage, before the self-reinforcing pathological state becomes deeply entrenched.

Several important limitations need to be acknowledged. Owing to the neonatal lethality of the dy^C^/dy^C^ mice, *in vivo* validation is not possible. Thus, it remains uncertain whether the therapeutic effects observed at the cellular level can translate into benefits for whole-organism factors such as drug bioavailability and tissue permeability, and the complexity of multiple organs cannot be fully mimicked in cell culture. Furthermore, all molecular analyses were conducted at birth (postnatal day 0), making it impossible to determine whether cAMP-PKA-DRP1 dysregulation precedes or follows the onset of α-DG glycosylation defects. Embryonic time-course profiling at multiple developmental stages would be required to establish the temporal hierarchy of these events and is planned as a focus of future investigation. Although significant functional recovery was achieved in cell experiments, complete repair might require additional interventions targeting parallel pathways or addressing underlying glycosylation defects. Developing conditional knockout models that allow mice to survive beyond the neonatal period would provide a means for *in vivo* validation of pharmacological interventions (Chan et al., 2010). Investigating whether variants in genes that cause DGPs besides *CRPPA* lead to similar pathway disruptions will verify the broad applicability of this mechanism. It is necessary to further explore the molecular mechanism connection between α-DG glycosylation defects and cAMP-PKA signaling pathway disorders, as understanding these connections may reveal new intervention targets. Combined therapies targeting glycosylation defects and mitochondrial dysfunction may produce synergistic benefits. Although this study verified the findings in human cells through patient-derived fibroblasts, future research using patient-derived induced pluripotent stem cells differentiated into muscle lineage cells will provide more validation platforms for verifying treatment plans for specific CRPPA variant-related diseases (Ortiz-Vitali and Darabi, 2019; Xia et al., 2018). Additionally, the *in vitro* validation was conducted in undifferentiated C2C12 myoblasts and dermal fibroblasts lacking mechanical stress, which may not fully recapitulate the core pathogenic mechanism in skeletal muscle. Future studies using differentiated myotubes under mechanical loading conditions will be important to address this limitation. Furthermore, the pharmacological rescue experiments relied on IBMX, a non-selective phosphodiesterase inhibitor that may activate multiple downstream effectors beyond PKA. Therefore, the mitochondrial rescue observed cannot be definitively attributed to PKA-mediated DRP1 Ser637 phosphorylation specifically. Genetic rescue approaches, such as expression of phosphomimetic DRP1 S637D or phospho-dead DRP1 S637A mutants, will be required to confirm pathway specificity in future studies.

In conclusion, this study successfully established a dy^C^/dy^C^ mouse model based on the patient-specific deletion of exons 6-9 of the *Crppa* gene, which accurately reproduced the severe phenotypes of human DGPs and provided new insights into the disease mechanism. The mouse model exhibited significant mitochondrial structural defects and reduced ATP levels, consistent with the mitochondrial abnormalities observed in patient muscle biopsies. RNA-sequencing analysis revealed dysregulation of the cAMP-PKA-DRP1 pathway, which regulates mitochondrial dynamics through DRP1 phosphorylation. In parallel, pharmacological activation of this pathway restored DRP1 phosphorylation, ATP production and mitochondrial membrane potential in both *Crppa* KD C2C12 cells and patient-derived fibroblasts. These findings challenge the traditional view of DGPs as a purely structural disease and reveal the significant contribution of metabolic dysfunction and mitochondrial dynamic abnormalities to the disease pathology. Identifying the cAMP-PKA-DRP1 pathway as a key pathogenic mechanism expands the therapeutic target landscape beyond glycosylation enhancement and gene replacement strategies. Although much work remains to translate these findings into clinical applications, this study lays a mechanistic foundation for the development of targeted therapies, which may provide a foundation for future therapeutic development for patients with *CRPPA*-related muscular dystrophy and other muscular dystrophies involving mitochondrial dysfunction.

## MATERIALS AND METHODS

### Ethics

The studies involving human participants were reviewed and approved by the Ethics Committee of Peking University First Hospital under approval number 2015[916]. They were conducted in accordance with the principles of the Declaration of Helsinki. Written informed consent to participate in this study was obtained from the participants’ legal guardians or next of kin. All animal experiments were approved by the Biomedical Ethics Committee of Peking University (Approval No. LA2022395), and the experimental procedures adhered to the guidelines outlined in the Guide for the Care and Use of Laboratory Animals (NIH publication, 8th edition, 2011).

#### Generation of a novel dy^C^/dy^C^ mouse model with deletion of exons 6-9 in *Crppa*

C57BL/6N mice were used to generate the *Crppa* knockout model via BIOCYTOGEN Corporation's EGE system based on CRISPR/Cas9 technology, targeting the *Crppa* gene (NCBI Gene ID 75847). Two sgRNAs were designed targeting introns 5 (5′-TCCCACTCTGGAGAATCCGG-3′) and 9 (5′-GAATTATGTCCCACGCCCTG-3′) of the *Crppa* gene. The sgRNAs and Cas9 mRNA were transcribed *in vitro* and microinjected into C57BL/6N fertilized eggs, which were then implanted into pseudopregnant females, yielding 54 F0 pups. Positive F0 mice identified by tail DNA genotyping were mated with WT mice to produce F1 offspring, and F2 mice were generated by mating heterozygous F1 mice.

Genomic DNA was extracted from toe clips of neonatal mice for genotyping. PCR amplification was performed using primers listed in Table S1 under the following conditions: 95°C for 3 min; 30 cycles of 95°C for 15 s, 62°C for 20 s, 72°C for 2 min; and a final extension at 72°C for 7 min. PCR products were separated by 2% agarose gel electrophoresis, whereby 506-bp fragments indicated WT, 717-bp fragments indicated homozygous (Hom) and the presence of both fragments indicated heterozygous (Het) genotypes (Fig. 1). The corresponding bands were excised from the gel, purified and subjected to Sanger sequencing for verification. Total RNA was extracted from quadriceps of 0-day-old mice (2-18 h postnatal, *n*=3 per group) and reverse transcribed using the RT system (A3500, Promega). RT-qPCR was performed to verify homozygous deletion of both N-terminal and C-terminal regions of *Crppa*. RT-qPCR conditions were as follows: 95°C for 5 min; 35 cycles of 95°C for 30 s, 56°C for 20 s, 72°C for 50 s; final extension at 72°C for 5 min. All primers are listed in Table S1. Results were quantified using the ΔΔCt method. Survival, weight and activity were monitored in 0-day-old WT, Het and Hom mice (*n*=20 per group).

#### Histopathology, immunofluorescence and electron microscopy

Lower limbs (biceps femoris, quadriceps), diaphragm, brain, heart and eyes were dissected from postnatal WT and dy^C^/dy^C^ mice. Tissues were paraffin embedded and sectioned at 7 μm for HE staining (Solarbio, Beijing, China), Masson's trichrome staining (Servicebio) and immunofluorescence staining. Primary and secondary antibodies are listed in Tables S2 and S3. HE- and Masson-stained sections were imaged using a Leica microscope (DFC295, Germany) with CaseViewer software. Immunofluorescence images were acquired using the Vectra Polaris Multispectral Tissue Imaging System (USA). For transmission electron microscopy (TEM), fresh skeletal muscle, cardiac, brain and ocular tissues from WT and dy^C^/dy^C^ mice were fixed in 3% glutaraldehyde. TEM sample preparation and imaging were performed by the Electron Microscopy Laboratory at Peking University First Hospital using a Tecnai G2 20 (FEI) microscope at 80 kV.

#### Western blot analysis

Total proteins were extracted from skeletal muscles or cultured cells using RIPA lysis buffer (Beyotime) supplemented with protease and phosphatase inhibitor cocktails (Roche). Protein concentrations were determined using a BCA protein assay kit (Thermo Fisher Scientific). Equal amounts of protein (30-50 μg) were separated by SDS-PAGE and transferred to PVDF membranes (Millipore). Membranes were blocked with 5% non-fat milk in TBST [Tris-buffered saline with Tween 20, containing 20 mM Tris-HCl (pH 7.4), 150 mM NaCl, and 0.1% Tween 20], incubated with primary antibodies overnight at 4°C, followed by horseradish peroxidase-conjugated secondary antibodies for 1 h at room temperature. Signals were detected using ECL reagent (Millipore) and visualized with a ChemiDoc imaging system (Bio-Rad). Band intensities were quantified using ImageJ and normalized to loading controls (β-actin or GAPDH). For membrane protein enrichment, 500 μg total protein was incubated with 50 μl WGA-conjugated agarose beads overnight at 4°C. Beads were washed three times with lysis buffer, and bound proteins were eluted by boiling in SDS sample buffer for western blot analysis. Laminin overlay assays were performed by incubating membranes with laminin-111 (5 μg/ml) for 3 h after protein transfer, followed by detection with anti-laminin antibody. All antibodies are listed in Tables S2 and S3.

#### RNA sequencing

Comprehensive RNA sequencing was performed using lower-limb skeletal muscles from 2-h-old WT (*n*=4) and dy^C^/dy^C^ (*n*=4) living mice. RNA extraction and sequencing were conducted at CapitalBio using TruSeq RNA Exome technology on an Illumina HiSeq X-Ten platform (2×150-bp paired-end reads), with samples processed blindly to minimize bias. The analysis pipeline included quality assessment (FastQC), preprocessing (FastP) and alignment to the mouse reference genome GRCm38 (Hisat2). Quality control ensured >90% gene coverage and monitored uniquely mapped reads and alignment to protein-coding sequences. StringTie was utilized for the detection of gene fusions, and principal component analysis was conducted to identify the primary sources of variation. In the differential expression analysis, featureCounts and StringTie were employed for quantification, while DESeq2 was used for normalization and the identification of differentially expressed genes (Love et al., 2014; Liu et al., 2021). Sample correlation was confirmed through Pearson analysis. Functional interpretation combined Gene Ontology, gene set enrichment analysis and KEGG pathway analysis using clusterProfiler, with validation through the GSVA package. Differential enrichment analysis used limma (significance: |log2FC|≥1, *P*<0.05). Results were visualized through hierarchical clustering with dynamic tree methods (Gene Ontology Consortium, 2006; Law et al., 2014).

#### *Crppa* KD C2C12 cells and *CRPPA*-related DGP patient-derived fibroblasts

C2C12 mouse myoblast cells (ATCC) were cultured in Dulbecco's modified Eagle medium (DMEM) supplemented with 10% fetal bovine serum (FBS) and 1% penicillin-streptomycin at 37°C in 5% CO_2_. For siRNA transfection, cells were seeded in six-well plates at 0.5-2×10^5^ cells/well and grown to 30-50% confluence. *Crppa*-specific siRNA and negative control siRNA (SyngenTech, Beijing, China) were transfected using Lipofectamine 2000 (Invitrogen) according to the manufacturer's protocol at a final concentration of 100 nM. Briefly, siRNA and Lipofectamine 2000 were each diluted in Opti-MEM (Thermo Fisher Scientific, 31985057), combined and incubated for 20 min before adding to cells in antibiotic-free medium. After 4-6 h, the medium was replaced with complete growth medium. KD efficiency was assessed by western blotting 48 h post transfection.

Primary human dermal fibroblasts were derived from skin biopsies of the patient with *CRPPA* variants and the unaffected control under informed consent and institutional ethics approval. Skin punch biopsies (3-5 mm) were obtained, minced and cultured as explants in DMEM with 20% FBS, 1% penicillin-streptomycin and 2 mM L-glutamine at 37°C in 5% CO_2_. Outgrown fibroblasts were passaged using 0.25% trypsin-EDTA, and cells at passages 3-8 were used for experiments. Fibroblasts were cultured in DMEM supplemented with 10% FBS and 1% penicillin-streptomycin at 37°C in 5% CO_2_.

#### Pharmacological rescue experiments

For drug treatment experiments, *Crppa* KD C2C12 cells (48 h post-transfection) and patient fibroblasts (70-80% confluence) were treated with IBMX (3-isobutyl-1-methylxanthine; Sigma-Aldrich, I5879) or 8-Br-cAMP (8-bromoadenosine 3′,5′-cyclic monophosphate; Sigma-Aldrich, B7880). Stock solutions (100 mM in DMSO or PBS, respectively) were stored at −20°C and diluted freshly in complete medium. Cells were treated with 400 μM IBMX or 8-Br-cAMP for 24, 48 or 72 h, with vehicle controls receiving 0.4% DMSO or PBS. Medium was not changed during treatment.

#### cAMP-PKA signaling pathway detection

RT-qPCR was performed to validate key genes in the cAMP-PKA signaling pathway. Total RNA was extracted using TRIzol reagent (Invitrogen) and reverse transcribed using the PrimeScript RT reagent kit (Takara). Quantitative PCR was conducted using SYBR Green Master Mix (Applied Biosystems) on a QuantStudio 5 Real-Time PCR System. Amplification conditions were 95°C for 10 min, followed by 40 cycles of 95°C for 15 s and 60°C for 1 min. Relative expression levels were calculated using the 2^(−ΔΔCt) method with *Gapdh* as the internal control. Primer sequences are provided in Table S1. Protein expression and phosphorylation status of PKA and DRP1 (Ser637) were assessed by western blot analysis as described above, using phospho-specific and total protein antibodies (Tables S2 and S3).

#### ATP detection

ATP levels were measured in mouse skeletal muscle tissues and cultured cells using an ATP Assay Kit (Beyotime, S0026) based on the luciferin-luciferase bioluminescence method. For tissue samples, ∼20 mg muscle was homogenized in 100-200 μl lysis buffer using a glass homogenizer on ice, then centrifuged at 16,201 ***g*** for 5 min at 4°C. Cells were lysed in 100 μl lysis buffer per well of a six-well plate, and lysates were clarified by centrifugation (12,000 ***g*** for 10 min at 4°C). Supernatants were collected and stored on ice. ATP detection working solution (100 μl) was added to each well of a white opaque 96-well plate and incubated for 3-5 min at room temperature to deplete background ATP. Subsequently, 20 μl sample or ATP standard was added, mixed by pipetting, and luminescence (relative light units) was measured immediately using a luminometer (Molecular Devices). Protein concentrations in lysates were determined using a BCA protein assay kit (Thermo Fisher Scientific), and ATP content was expressed as nmol/mg protein.

#### JC-1 staining for mitochondrial membrane potential assessment

Mitochondrial membrane potential was assessed using a JC-1 Mitochondrial Membrane Potential Detection Kit (BestBio, BB-4105). After drug treatment, C2C12 cells and human fibroblasts were washed twice with PBS and incubated with JC-1 staining working solution for 20 min at 37°C in 5% CO_2_. Cells were then washed twice with cold PBS and immediately observed under a fluorescence microscope (Olympus, IX73). JC-1 monomers in depolarized mitochondria emitted green fluorescence (excitation 490 nm/emission 530 nm), while JC-1 aggregates in polarized mitochondria with intact membrane potential emitted red fluorescence (excitation 550 nm/emission 590 nm). The red/green fluorescence intensity ratio was quantified using ImageJ software from at least five random fields per condition. Decreased red/green ratios indicate mitochondrial depolarization and membrane potential loss.

#### Statistical analysis

Statistical analyses were performed using GraphPad Prism 10 (GraphPad Software, USA). Data are presented as mean±s.d. Unpaired two-tailed Student's *t*-tests were used for comparisons between two groups. One-way ANOVA followed by Tukey's multiple comparison test was used for comparisons among three or more groups. Two-way ANOVA was employed for experiments with two independent variables (e.g. genotype and treatment). Kaplan–Meier survival curves were generated for lifespan analysis. Western blot band intensities, tissue areas, cell numbers in HE/Masson staining and immunofluorescence intensities were quantified using ImageJ software. *P*<0.05 was considered significant. All experiments were performed with at least three biological replicates unless otherwise specified.

## Supplementary Material

10.1242/dmm.052890_sup1 Supplementary information
