# Supplementary material for: Mitochondrial defects in the cAMP-PKA-DRP1 pathway in a Crppa deletion model of dystroglycanopathy
Source: Dis Model Mech. 2026 Sep 17;19(9):dmm052890. doi: 10.1242/dmm.052890 (PMC13617977; doi:10.1242/dmm.052890)
Supplement: Supplementary file 1 [file dmm-19-052890-s1.pdf]

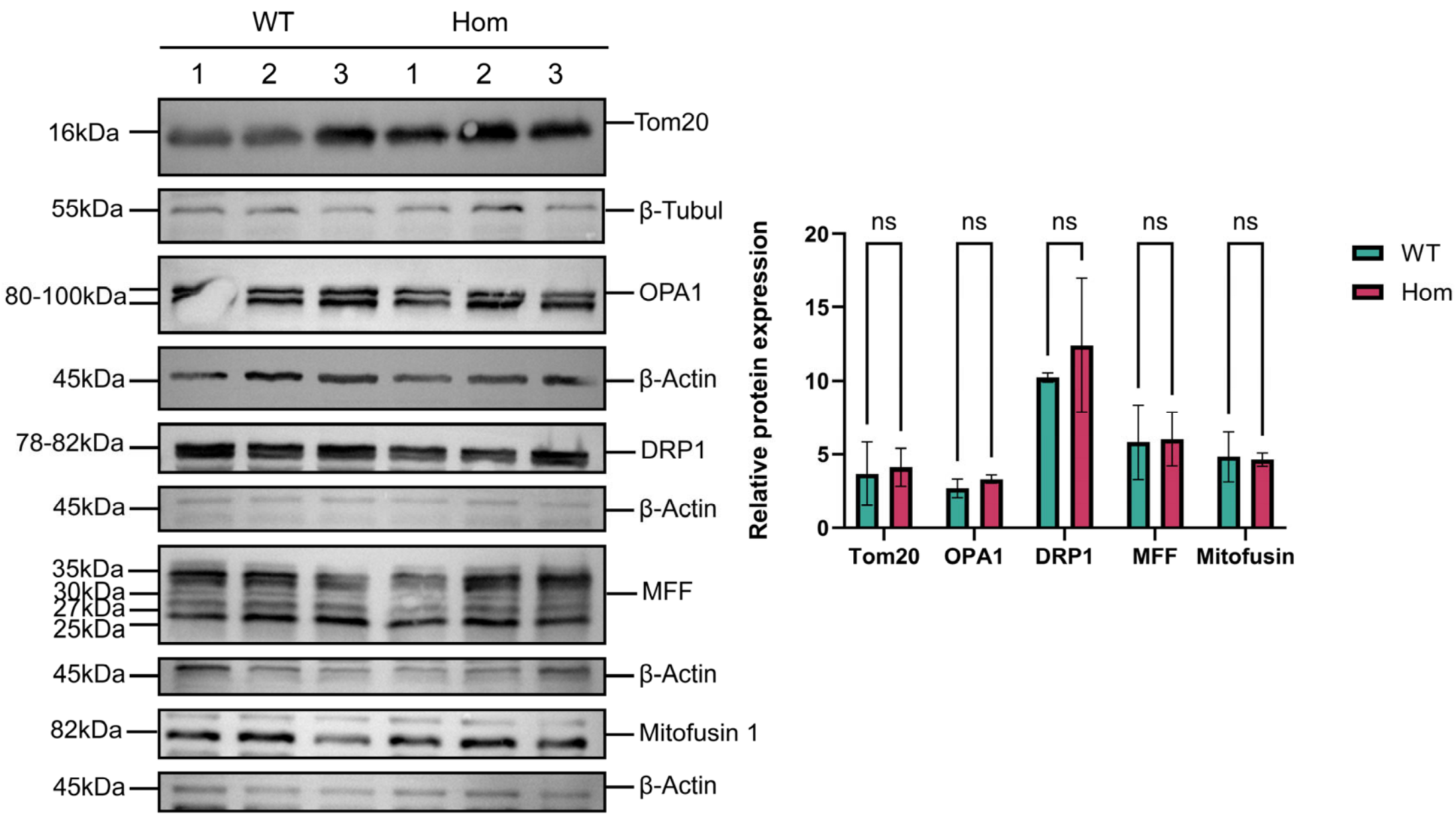

Fig. S1. Western blot analysis of mitochondrial dynamics.

Tom20, which is localized to the outer mitochondrial membrane, facilitates the import of precursor proteins across the mitochondrial outer membrane by recognizing the presequence. OPA1, a mitochondrial inner membrane-associated dynamin-related protein, plays a critical role in inner membrane fusion and cristae maintenance. DRP1 is a member of the dynamin superfamily of GTPases. MFF is a tail-anchored protein that resides within the outer mitochondrial membrane and is part of the mitochondrial fission complex. MFF participates in mitochondrial fission by serving as one of multiple receptors for the GTPase Drp1. Mitofusins are mitochondrial transmembrane GTPases that function to regulate mitochondrial fusion, a process that occurs in concert with mitochondrial division and is necessary for the maintenance of structural and genetic mitochondrial integrity. The results indicated no differences in the expression levels of mitochondrial dynamics-related proteins between Hom and WT mice ($n = 3$, ns, $p > 0.05$, by two-way ANOVA test). DRP1, Dynamin-related protein 1; MFF, Mitochondrial fission factor; WT, wild-type; Hom, homozygous.

Table S1. Primer sequences.

Primer names	sequences
Crppa-C forward （RT-qPCR）	5'-ATGGAAAGCCTGGTGTGGATT-3'
Crppa -C reverse （RT-qPCR）	5'-GCTTCTGCTCATCCTGTGAGT-3'
Crppa -N forward （RT-qPCR）	5'-AGGAACATGGGTGTAGTGATTT-3'
Crppa -N reverse （RT-qPCR）	5'-AGCTGCACACAGGTCTTGTT-3'
GAPDH forward （RT-qPCR）	5'-GGCTGCCCAGAACATCATCCCT-3'
GAPDH reverse （RT-qPCR）	5'-ATGCCTGCTTCACCACCTTCTTG-3'
Jun-forward （RT-qPCR）	5'-TGGGCACATCACCACTACAC-3'
Jun-reverse （RT-qPCR）	5'-TCTGGCTATGCAGTTCAGCC-3'
Sox9-forward （RT-qPCR）	5'-GTGCAAGCTGGCAAAGTTGA-3'
Sox9-reverse （RT-qPCR）	5'-TGCTCAGTTCACCGATGTCC-3'
Hcar2-forward （RT-qPCR）	5'-CTTCCTACCCAGTGTGGCTG-3'
Hcar2-reverse （RT-qPCR）	5'-CAGGTCCACCGAGGAGTAGA-3'
Mc2r-forward （RT-qPCR）	5'-TTCTCAGTCATCTTGCCGAG-3'
Mc2r-reverse （RT-qPCR）	5'-GCTTGCTGGTTGAGGGTAGA -3'
Fos-forward （RT-qPCR）	5'-TACTACCATTCCCCAGCCGA-3'
Fos-reverse （RT-qPCR）	5'-GCTGTCACCGTGGGGATAAA-3'
Calm4-forward （RT-qPCR）	5'-ACTAAGGAGGAGGTCGCTGA-3'
Calm4-reverse （RT-qPCR）	5'-TTCCTCGACGCTGATATGGC-3'
Crppa-F(Genotyping)	TTTCCTCTTTGGTCACCATGGTTTC
Crppa-R1(Genotyping)	TCAATACAGTGCAGTTCCTGTTTGG
Crppa-R2(Genotyping)	AAACCACTCCAAAAGCAACTTCAG

Table S2. Primary antibodies.

Primary antibody names	company identification （Catalog#)	Dilution
Rabbit monoclonal CRPPA	SinoBiological(BDF20)	WB 1:500
Mouse monoclonal IIH6	Millipore(05-593)	WB 1:1000 IF 1:200
Rabbit monoclonal β-DG	Abcam(ab43125)	WB 1:1000
Rabbit polyclonal β-DG	Santa Cruz (sc-23585)	IF 1:1000
Rat monoclonal α-DG core(3D7)	Millipore(MABN2515)	WB 1:1000
Sheep monoclonal α-DG core(Dystroglycan)	R&D systems(AF6868)	WB 1:1000
Rabbit monoclonal Laminin	Abcam(ab11575)	Laminin Overlay 1:1000
Rabbit monoclonal GAPDH	Cell Signaling Technology(#5174)	WB 1:2000
Rabbit monoclonal Tom20	Cell Signaling Technology(#42406)	WB 1:1000
Rabbit monoclonal OPA1	Cell Signaling Technology(#80471)	WB 1:1000
Rabbit monoclonal DRP1	Cell Signaling Technology(#8570)	WB 1:1000
Rabbit monoclonal Phospho-DRP1 (Ser637)	Cell Signaling Technology(#6319)	WB 1:1000
Rabbit monoclonal MFF	Cell Signaling Technology(#84580)	WB 1:1000
Rabbit monoclonal Mitofusin-1	Cell Signaling Technology(#14739)	WB 1:1000
Rabbit monoclonal β-actin	Cell Signaling Technology(#4970)	WB 1:1000
Rabbit monoclonal β-Tubulin	Cell Signaling Technology(#2146)	WB 1:1000
Rabbit monoclonal Phospho-PKA C	Cell Signaling Technology(#4781)	WB 1:1000
Rabbit monoclonal PKA C-α	Cell Signaling Technology(#4782)	WB 1:1000

Table S3. Secondary antibodies.

Secondary antibody names	company identification （Catalog#)	Dilution
Goat Anti Rabbit IgG-HRP	Gene-Protein Link(P03S02)	WB 1:1000
Goat Anti mouse IgG-HRP	Gene-Protein Link(P03S01)	WB 1:1000
Rabbit Anti rat IgG-HRP	Gene-Protein Link(P03S10)	WB 1:5000
Rabbit Anti sheep IgG H&L	Abcam(ab6747)	WB 1:2000
Goat anti-mouse-IgG Alexa Fluor 488	Thermo Fisher Scientific (A-11001)	IF 1:200
Goat anti-rabbit-IgG Alexa Fluor 555	Thermo Fisher Scientific (A-21428)	IF 1:200

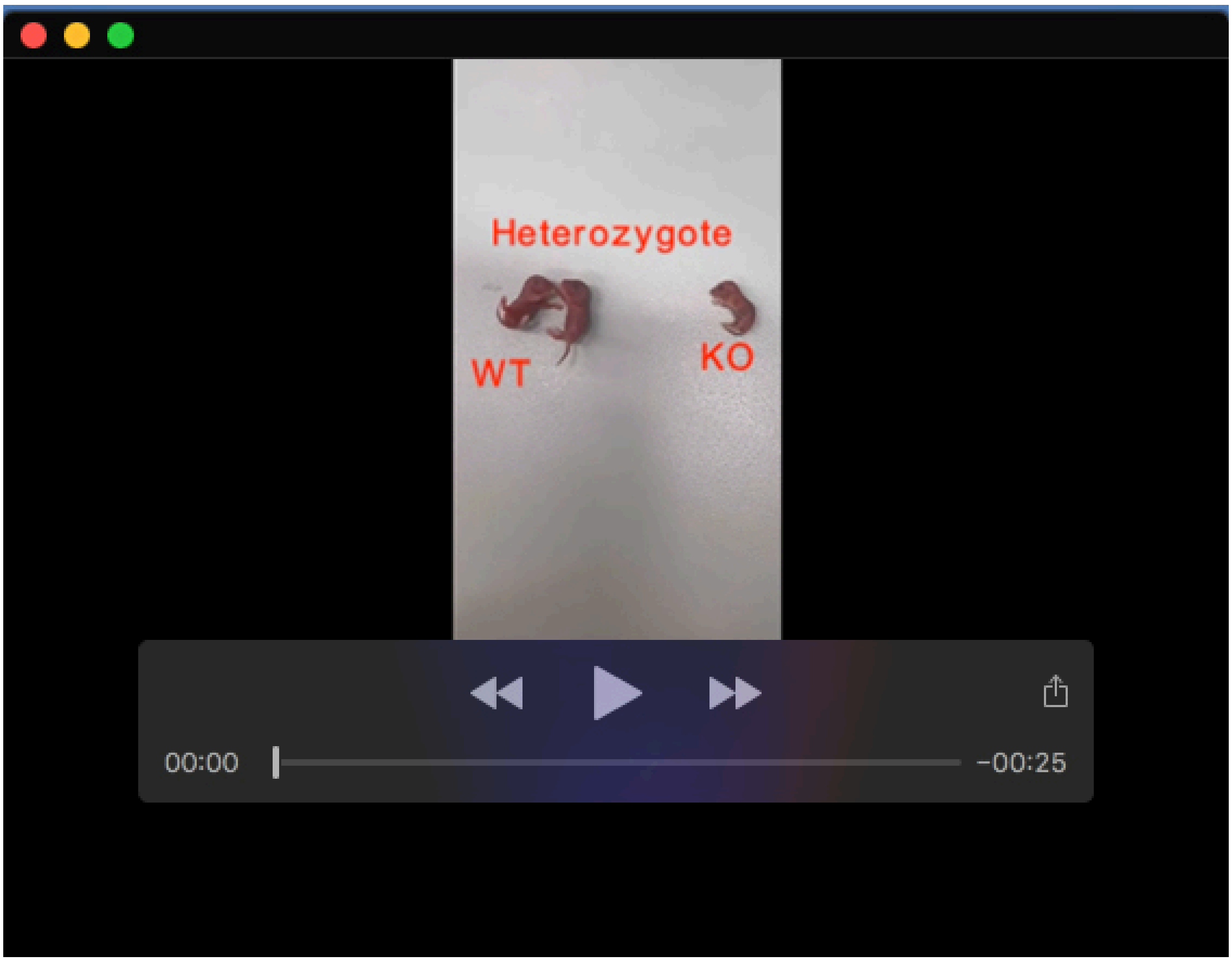

Movie 1. Activity of WT, heterozygous and KO (dy^C/dy^C) mice.
